# NMDA and PACAP Receptor Signaling Interact to Mediate Retinal-Induced SCN Cellular Rhythmicity in the Absence of Light

**DOI:** 10.1371/journal.pone.0076365

**Published:** 2013-10-01

**Authors:** Ian C. Webb, Lique M. Coolen, Michael N. Lehman

**Affiliations:** 1 Department of Neurobiology and Anatomical Sciences, University of Mississippi Medical Center, Jackson, Mississippi, United States of America; 2 Department of Physiology & Biophysics, University of Mississippi Medical Center, Jackson, Mississippi, United States of America; 3 Department of Anatomy and Cell Biology, University of Western Ontario, London, Ontario, Canada; Kent State University, United States of America

## Abstract

The “core” region of the suprachiasmatic nucleus (SCN), a central clock responsible for coordinating circadian rhythms, shows a daily rhythm in phosphorylation of extracellular regulated kinase (pERK). This cellular rhythm persists under constant darkness and, despite the absence of light, is dependent upon inputs from the eye. The neural signals driving this rhythmicity remain unknown and here the roles of glutamate and PACAP are examined. First, rhythmic phosphorylation of the NR1 NMDA receptor subunit (pNR1, a marker for receptor activation) was shown to coincide with SCN core pERK, with a peak at circadian time (CT) 16. Enucleation and intraocular TTX administration attenuated the peak in the pERK and pNR1 rhythms, demonstrating that activation of the NMDA receptor and ERK in the SCN core at CT16 are dependent on retinal inputs. In contrast, ERK and NR1 phosphorylation in the SCN shell region were unaffected by these treatments. Intraventricular administration of the NMDA receptor antagonist MK-801 also attenuated the peak in SCN core pERK, indicating that ERK phosphorylation in this region requires NMDA receptor activation. As PACAP is implicated in photic entrainment and is known to modulate glutamate signaling, the effects of a PAC_1_ receptor antagonist (PACAP _6-38_) on SCN core pERK and pNR1 also were examined. PACAP _6-38_ administration attenuated SCN core pERK and pNR1, suggesting that PACAP induces pERK directly, and indirectly via a modulation of NMDA receptor signaling. Together, these data indicate that, in the absence of light, retinal-mediated NMDA and PAC_1_ receptor activation interact to induce cellular rhythms in the SCN core. These results highlight a novel function for glutamate and PACAP release in the hamster SCN apart from their well-known roles in the induction of photic circadian clock resetting.

## Introduction

The hypothalamic suprachiasmatic nucleus (SCN) is a central clock essential for circadian (i.e., ∼24 hr) rhythms in a wide variety of behavioral and physiological endpoints, as well as for the ability of light to entrain rhythms to the twenty-four hour world [1], [2], [3]. At the tissue level, SCN neurons show regional heterogeneity with regard to both neuropeptide expression and cellular rhythmicity [4], [5], [6]. In rodents, phosphorylated extracellular-regulated kinase (pERK; also, known as MAP kinase) defines two subpopulations of endogenously rhythmic SCN cells whose peak expression is in anti-phase to each other. During the subjective day, there is increased pERK in the dorsomedial “shell” region, whereas during the subjective night, pERK is increased in the ventrolateral “core” portion of the nucleus [7], [8], [9]. While most cellular rhythms are intrinsic to the SCN [10], [11], [12], the endogenous rhythm in SCN core pERK is dependent upon the eye as it is selectively abolished by enucleation [9]. Hence, retinal input induces cellular rhythmicity in a sub-compartment of the master circadian clock, even in the absence of light.

The role of the eye in photic entrainment is well-established. Synchronization of circadian rhythms to the external light/dark cycle is largely mediated by intrinsically photoreceptive retinal ganglion cells (ipRGCs) that project to the SCN via the retinohypothalamic tract (RHT) [13], [14], [15], [16]. Photic stimulation at night results in an induction of *period* gene expression in the SCN [17], [18], [19] and a resetting of the molecular clock mechanism that mediates circadian timing [20]. Both glutamate and pituitary adenylate cyclase activating peptide (PACAP) are co-stored in retinal terminals [21], [22], [23] and have been implicated in SCN photic signal transduction [24]. NMDA receptor agonists, for example, can induce photic-like phase shifts of circadian rhythms, while antagonists can block light induced clock resetting [25], [26], [27]. Similarly, PACAP, when administered in nanomolar concentrations, induces photic-like phase shifts [28], [29], [30], and can increase SCN pERK and *period1* expression [30], [31]. PACAP also modulates the postsynaptic response to glutamate in the SCN and other brain regions [28], [32], [33] and thus can exert at least some of its effects via a glutamatergic mechanism.

Here, we sought to elucidate the RHT output signals necessary for the rhythm in SCN core ERK phosphorylation that occurs in the absence of light. Retinal efferents innervate the core region [34], [35] and directly contact core pERK cells [9], suggesting that pERK may be induced directly by RHT neurotransmitter release. Given the roles of glutamate and PACAP in photic entrainment, it was hypothesised that both NMDA and PAC_1_ receptor activation, induced via inputs from the eye, interact to generate rhythmic SCN core ERK phosphorylation in the absence of light. This hypothesis was tested by examining the association between phosphorylation of the NR1 NMDA receptor subunit (pNR1) [36], [37] and rhythmic pERK in the SCN core region. The effects of enucleation and intraocular tetrodotoxin administration on SCN core NMDA receptor and ERK activation rhythms also were examined. Furthermore, using pharmacological manipulations it was tested if activation of NMDA or PAC_1_ receptors is required for retinal-mediated pERK and pNR1 in the SCN core region.

## Materials and Methods

### Animals

Adult male Syrian hamsters (Charles River, Montreal, QC, CAN) were used for all experiments. Animals were singly housed in running wheel cages and entrained to a 14:10 light/dark cycle (∼350lux L/0 lux D) for at least one week prior to the onset of experiments. Food and water were available *ad libitum*. Wheel revolutions were continuously monitored via magnetic switches and recorded using VitalView software (Mini Mitter, Bend, OR, USA). All procedures were approved by the Animal Use Subcommittee at the University of Western Ontario and the University Committee on Use and Care of Animals at the University of Michigan.

### Enucleation surgery

Animals were left undisturbed (n = 32) or were bilaterally enucleated (n = 34) under isoflurane anesthesia. Following removal of the eye, the sockets were filled with Gelfoam to reduce bleeding and the eyelids were sutured shut. Ketoprofen (5 mg/kg, s.c.) was administered as an analgesic. Following surgery, all animals were housed under constant darkness (DD) for one week prior to tissue collection. Clocklab was used to predict activity onsets (designated circadian time [CT] 12, by convention) and to calculate the time at which brains were to be collected. Animals were transcardially perfused at CT0, 4, 8, 12, 16, and 20 (n = 5–6/time pt. per condition), brains were collected, and sections through the SCN stained for both pERK and pNR1 using the immunofluorescent technique as described below.

### Intraocular TTX Administration

Following one week in DD, 2 µl of citrate buffer vehicle (20 mM, pH 4.8 with 15 µg/ml methylene blue added to aid with injection visualization; n = 31) or tetrodotoxin (TTX, 2 µg/µl in vehicle, Sigma-Aldrich, St. Louis, MO, USA; n = 31) were injected into the vitreous chamber of each eye at the level of the ora serrata. Injections were administered with a cuffed needle attached to a 10 µl Hamilton syringe that allowed for a 1 mm projection of the needle into the eye. The syringe was held in place for 1 min after completion of volume ejection to allow for adequate diffusion. All procedures were carried out under dim red light and the animals were anesthetized with isoflurane throughout. Injections were either during the early subjective day (CT3) with perfusion as described below at CT4, 8, and 12, or during the early subjective night (CT15) with perfusion at CT16, 20, and 0 (n = 5–6/time pt. per condition). Sections through the SCN were stained for both pERK and pNR1 using the immunofluorescent technique as described below.

**Figure 1:**
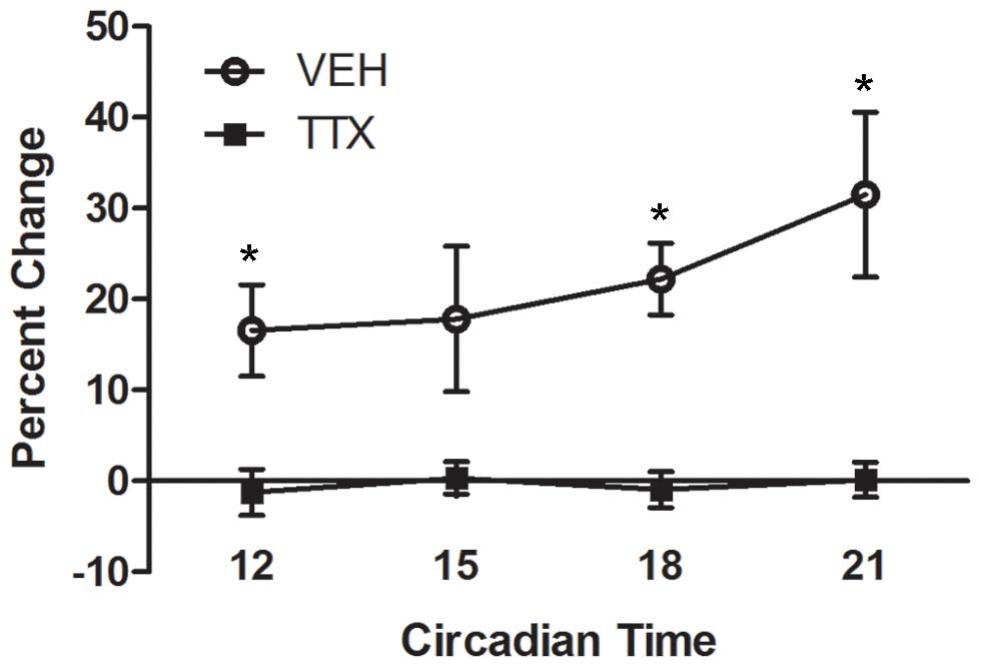
Inhibition of pupillary light reflex by intraocular tetrodotoxin (TTX) over a 10 hour period. Shown is the percent change in the pupil diameter to eye diameter ratio induced by a 30s blue light pulse following intraocular administration of vehicle (VEH) or TTX at circadian time 11. * = p<0.05 vs. TTX.

To ensure that TTX administered at this dose inhibits retinal output over the time course of our experiments, we assessed the pupillary light reflex (PLR) following intraocular administration. Animals were housed under DD for 2 days and at CT11 were administered either vehicle (n = 6) or TTX (n = 5) as described above. Beginning one hour after administration and continuing every 3 hours for a 9 hour period, animals were briefly anaesthetized with isoflurane, and a digital video camera fitted with a +2 diopter lens and an infrared filter was used to capture an image of the eye (in a separate pilot study we determined that isoflurane did not significantly influence the PLR). Hamsters were then subjected to a 30s light pulse (∼470 nm peak, 75 nm half-peak width) and the eye was re-imaged. The photos were imported into ImageJ (NIH), a pupil diameter to eye diameter ratio was determined for each animal prior to and after the light pulse, and the percent change in pupil to eye ratio was calculated. Analysis revealed that intraocular TTX administration at the current dose significantly inhibited the PLR for at least 10 hours following injection (**Fig. 1**).

To further establish that TTX transiently attenuates pERK and does not induce permanent ocular damage, an additional group of animals were injected with TTX at CT15, perfused one day, two days, or one week later at CT16 (n = 3–5 per group), and sections were immunostained for pERK using the immunoperoxidase technique as outlined below.

### Tissue Collection

Hamsters were deeply anesthetized with sodium pentobarbital (∼300 mg/kg, i.p.) and perfused transcardially with saline followed by 150 ml of cold 4% paraformaldehyde in 0.1 M phosphate buffer (PB). All perfusions were performed under dim red light (∼ 1 lux). Brains were removed, postfixed for 1 hr, equilibrated in 20% sucrose in PB, and stored at 4°C. Coronal sections (35 µm) were cut on a freezing stage microtome, collected into four parallel series, and stored in cryoprotectant solution [38] at –20°C until immunohistochemical processing.

### Immunofluorescent Staining and Analysis

Sections from the SCN of intact, enucleated, vehicle- and TTX-treated animals were immuno-labeled for pERK and pNR1 using fluorescent techniques. All incubations were carried out at room temperature with free-floating sections and the tissue was extensively washed with 0.1 M phosphate buffered saline (PBS) between steps. Immunological reagents were diluted in incubation solution consisting of PBS with 0.4% Triton X-100 (Sigma-Aldrich) and 0.1% bovine serum albumin (Fisher Scientific, Ottawa, ON, Canada). To reduce non-specific staining, the tissue was first washed in 0.01% sodium azide (Fisher Scientific) in PBS (2×45 min), then exposed to 1% H_2_O_2_ (Fisher Scientific) in PBS (10 min), and subsequently blocked in incubation solution for 1 hr. Next, the tissue was incubated with a rabbit polyclonal antibody specific for the phosphorylated forms of ERK 1 and 2 (1∶1000, phospho-p44/p42 MAP Kinase, cat. # 9101L, Cell Signaling Technology, Inc., Danvers, MA, USA, 17hr) followed by biotinylated goat anti-rabbit IgG (1∶400, Jackson ImmunoResearch Laboratories, Inc., West Grove, PA, USA, 1hr), HRP-conjugated avidin-biotin complex (1∶1000 in PBS, Vector Laboratories, Burlingame, CA, USA, 1 hr), biotinylated tyramide (1∶250 with 0.003% H_2_O_2_ in PBS, PerkinElmer, Waltham, MA, USA, 10 min), and Alexa 488 conjugated streptavidin (1∶200 in PBS, Molecular Probes, Carlsbad, CA, USA, 30 min). The sections were subsequently exposed to a rabbit anti-pNR1 IgG that detects single or dual phosphorylation at Ser896 and Ser897 (1∶5000, overnight, cat. # 06-641, Upstate Biotech, Lake Placid, NY, USA) followed by CY3 conjugated donkey anti-rabbit IgG (1∶400, Jackson Immunoresearch, 30min). Sections were mounted onto plus-charged glass slides, dried, and coverslipped with an aqueous mounting medium (Gelvatol) containing an anti-fading agent [1,4-diazabicyclo(2,2)octane (DABCO); 50 mg/ml, Sigma-Aldrich, prepared as described previously [39]. These antibodies have been extensively characterized and yield appropriate bands on western blot analysis [36], [37], [40], [41]. Omission of the primary antibodies resulted in a lack of staining.

**Figure 2:**
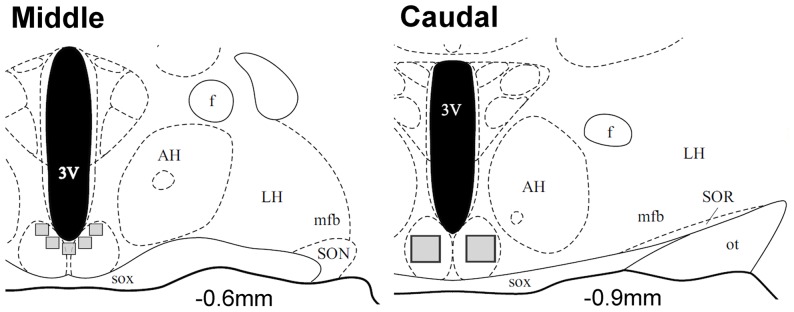
Approximate counting box placement for quantification of pERK and pNR1. The smaller boxes outline areas analyzed in the shell at the middle (–0.6 mm relative to bregma) level and the larger those in the core at the caudal (–0.9 mm) level. SOX: optic chiasm, 3V: 3^rd^ ventricle, AH: anterior hypothalamus, LH: lateral hypothalamus, SON: supraoptic nucleus, f: fornix, mfb: medial forebrain bundle, ot: optic tract. Modified from Morin & Wood, 2001.

Images of pERK and pNR1 immunofluorescent staining were captured for two sections comprising the middle and caudal SCN of each animal using Neurolucida software (MicroBrightfield Bioscience, Williston, VT, USA) and a digital camera (Microfire A/R, Optronics, CA, USA) attached to a microscope (DM500B, Leica Microsystems, Wetzlar, Germany). Identification of the rostral/caudal level and the core/shell regions of each section was based on various landmarks including the shape of the optic chiasm and the supraoptic nucleus (SON), as determined previously [9]. Counting boxes were placed on the images to delineate the boundaries of the shell at the middle level and the core at the caudal level. Five 80 µm x 80 µm boxes at the middle level and two 160 µm W x 180 µm L boxes at the caudal level were used to quantify staining (**Fig. 2**). Single- and double-labeled cells located within these boxes were counted by an observer blind to experimental condition. Data are expressed as the mean number of cells per animal +/– SEM.

**Figure 3:**
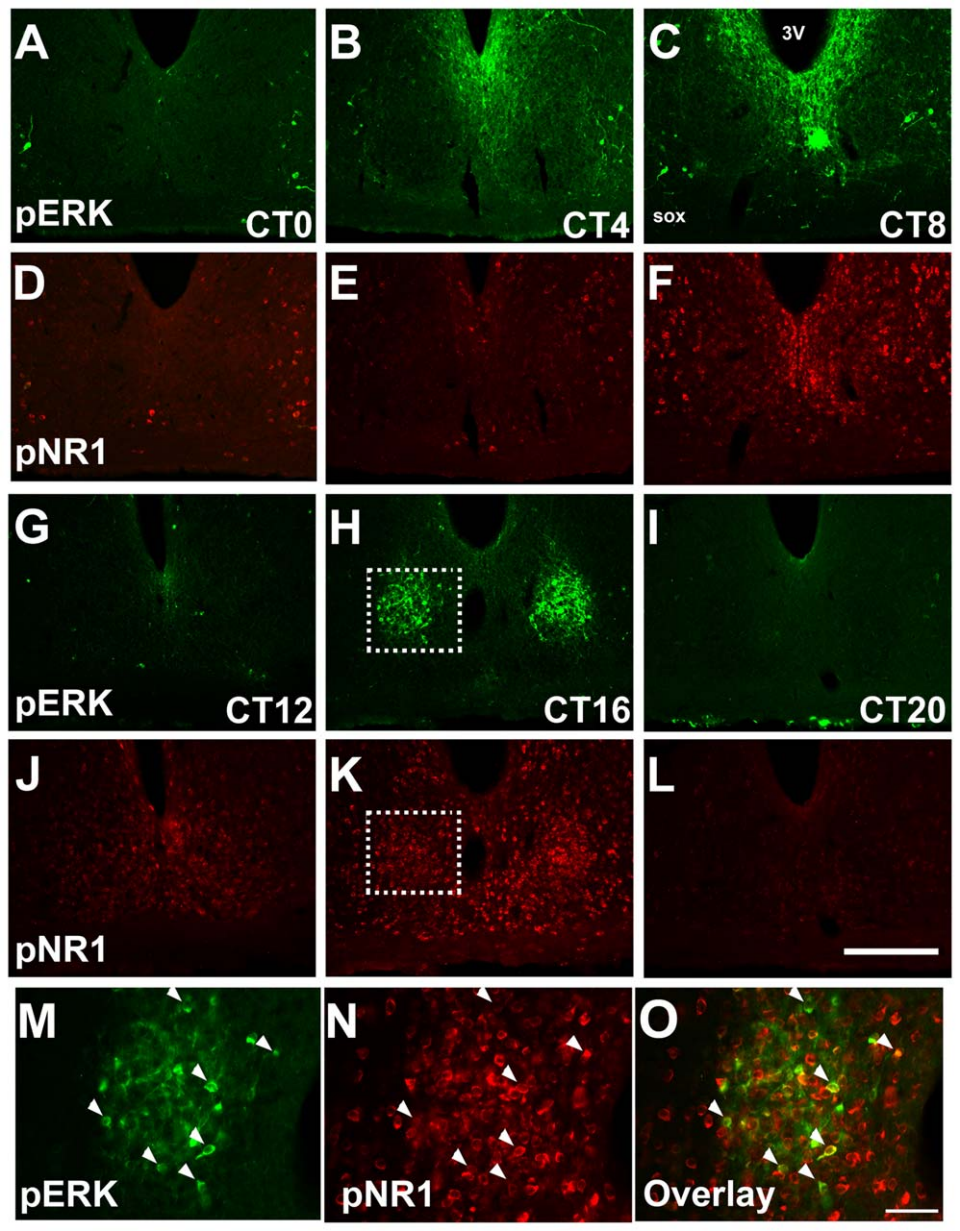
Circadian variation in pERK and pNR1 immunoreactivity in the SCN. A-L: Shown are representative images of pERK (green) and pNR1 (red) immunostaining in intact animals across the circadian day. The white boxes outline the area enlarged in panels M-O. Scale bar = 200 µm. M-O: Colocalization of pERK and pNR1 immunoreactive cells in the SCN core of an intact animal perfused at CT16. Arrows indicate some examples of double-labelled cells. Scale bar  =  50 µm.

As pERK immunostaining in the shell region is dense making it difficult to distinguish individual cells (see **Fig. 3C**), densitometry analysis also was used for quantification in this area. Images of one middle SCN section per animal were captured as outlined above. Image J (NIH) was used to apply a fixed pixel intensity threshold to the images. The percent area containing pixels above the fixed threshold was averaged over the five shell counting boxes for each animal.

Two-way or one-way ANOVAs followed by Bonferroni post-hoc comparisons were used to analyze treatment and time-of-day effects. When normality or equal variance tests failed, the non-parametric Kruskal-Wallis test followed by Dunn’s multiple comparisons were used. The significance level was set at *p* = 0.05 for all tests.

### Intraventricular Drug Administration

Under sodium pentobarbital anaesthesia (80 mg/kg, i.p.), animals were implanted with a guide cannula (7.0 mm, 22 g, Plastics One, Roanoke, VA, USA) aimed at the 3^rd^ ventricle (stereotaxic coordinates: 1.0 mm anterior and 0.0 mm lateral to bregma, and 7.0 mm ventral to the skull surface). The guide cannulae were cemented to the skull with dental acrylic and screws, and dummy cannulae were inserted to prevent blockage. Ketoprofen (5 mg/kg, s.c.) was administered as an analgesic.

Following surgery, the animals were placed into DD for 7–8 days and at CT15 were subjected to one of two pharmacological treatments: 1) the non-competitive NMDA receptor channel blocker MK-801 (0.5 µl of 8.9 mM, n = 5 or 0.5 µl of 17.8 mM, n = 5; Sigma-Aldrich) or vehicle (0.5 µl sterile saline, n = 8) or 2) the PAC_1_ receptor antagonist PACAP_6–38_ (2.5 µl of 0.1 mM, n = 5; Bachem, Torrance, CA, USA) or vehicle (2.5 µl sterile saline, n = 5). These pharmacological agents when administered near the current doses have previously been reported to attenuate photic-induced phase shifts in hamsters [26], [42].

The injections were given via an injector cannula (projecting 1 mm beyond the tip of the guide cannula) attached to a syringe pump (Harvard Apparatus, Holliston, MA, USA) over a two minute period at flow rates of 0.25 µl/min (MK-801), or 1.25 µl/min (PACAP_6–38_). The injector cannula was left in place for an additional minute to allow for adequate diffusion. One hour following the injections (i.e., CT16), brains were collected as described above.

### Immunoperoxidase Staining and Analysis

SCN sections from animals treated with the various pharmacological agents were stained for pERK using an avidin-biotin-immunoperoxidase technique. All general procedures were as described above. The sections were incubated with polyclonal antibody specific for pERK 1/2 (1∶1000, 17hr, Cell Signaling, cat. # 9101L), biotin-conjugated goat anti-rabbit IgG (1∶500, Vector Laboratories, 1 hr), and HRP-conjugated avidin-biotin complex (1∶1000 in PBS, Vector Laboratories, 1 hr). Staining was visualized via immersion in 0.02% diaminobenzidine (Sigma-Aldrich), 0.08% nickel sulfate, and 0.01% H_2_O_2_ in PB for 10 min. The sections were mounted onto plus charged glass slides, dehydrated in a series of graded alcohols, cleared in CitriSolv (Fisher Scientific), and cover slipped with dibutyl phthalate xylene (DPX; Electron Microscopy Sciences, Hatfield, PA, USA).

The tissue was examined to verify correct cannula placement. In some animals, the ventricle was slightly enlarged, but this occurred evenly across all treatment groups and did not influence pERK expression.

Images of one-two caudal SCN sections per animal were captured with a digital camera (Magnafire, Optronics, CA, USA) attached to a microscope (DM500B, Leica Microsystems) and Image J was used to apply a fixed pixel intensity threshold to all images. An oval area of analysis (140 µm W x 240 µm H) was placed over the core pERK region and the percent of the area containing pixels above threshold was calculated for each animal. As a control, pERK immunostaining was also quantified in the SON. In this area, a circular area of analysis (220 µm diameter) was used. Data are expressed as the average per animal +/− SEM and were analyzed using one-way ANOVA followed by Bonferroni comparisons, or independent t-tests as appropriate.

A separate series of SCN sections from MK-801, PACAP_6-38_, and vehicle treated animals were also labeled for pNR1 using a rabbit anti-pNR1 antibody (1∶100,000, Upstate Biotech, cat. # 06-641) and the avidin-biotin-immunoperoxidase technique as described above. Numbers of pNR1 cells in the core region from one caudal SCN section per animal were counted in a 180 µm H x 160 µm W counting box with the aid of a drawing tube attached to a microscope (DM500B, Leica Microsystems). Data are expressed as the average per animal +/− SEM and were analyzed using one-way ANOVA followed by Bonferroni post-hoc comparisons, or independent t-tests as appropriate.

## Results

### SCN core pERK and pNR1 cells colocalize and their rhythms coincide

To examine the temporal associations between SCN core pERK and pNR1, and to determine the extent of colocalization of these markers, analysis of single and double-labeled pERK and pNR1 immunoreactive neurons was carried out in intact untreated animals across the circadian day. The number of SCN core pERK cells varied in a circadian fashion (H [5, N =  32]  = 13.6, *p* = 0.018) with a peak at CT16 and nadirs at CT4 (*p*<0.05 vs. CT16) and CT8 (*p*<0.05 vs. CT16; **Figs. 3A-C & G-I** **, and** **4A**), as observed previously [7], [8], [9]. Similarly, the number of pNR1 cells in the core region varied across the day (F [5], [26]  = 4.53, *p* = 0.004) with a peak at CT16 and nadirs at CT0 (*p* = 0.0035 vs. CT16) and CT4 (*p* = 0.0024 vs. CT16; **Figs. 3D-F & J-L** **, and** **4B**). Analysis of double-labelled cells revealed that the vast majority of core pERK neurons observed at CT16 colocalized with pNR1 (93.5 +/− 4.6% per animal, **Fig. 3M-O**), whereas fewer pNR1 cells (12.7 +/− 2.7%) at this time point colocalized with pERK.

**Figure 4:**
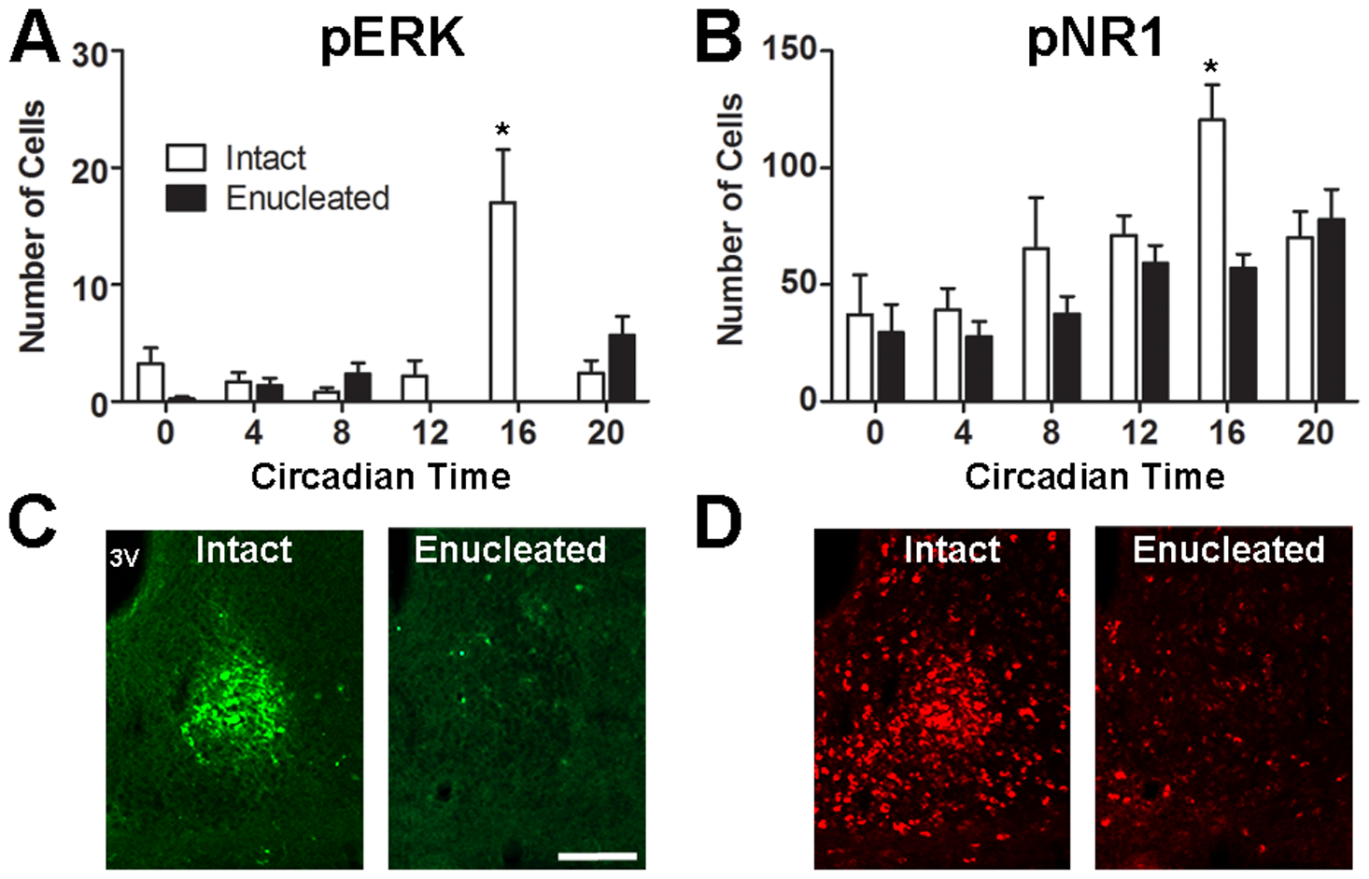
The effect of enucleation on ERK and NR1 phosphorylation rhythms in the SCN core. Shown are the numbers of pERK (A) and pNR1 (B) cells in the SCN core across the circadian day in intact and enucleated animals. Also shown are representative images of SCN core pERK (C) and pNR1 (D) immunostaining from intact and enucleated animals at CT16. Scale bar  =  100 µm. * = p<0.05 vs. enucleated animals at CT16.

### Enucleation and intraocular TTX administration attenuate the peak in SCN core pERK and pNR1 rhythms

Enucleated animals were examined to determine if pNR1, like pERK, is attenuated by removal of the eye. For pERK, there were overall effects of time (F[5], [55]  = 7.47, *p*<0.0001), enucleation (F[1], [55]  = 11.95, *p* = 0.001), and a significant interaction (F[5], [55]  = 11.91, *p*<0.0001), while significant main effects of time (F[5], [54] =  7.42, p<0.0001) and enucleation (F[1], [54]  = 8.04, p = 0.0064) were observed for pNR1. The peak in the number of SCN core pERK cells at CT16 was eliminated by enucleation (*p*<0.0001; **Fig. 4A & C**), in agreement with previous results [9]. Similarly, removal of the eye significantly decreased the peak in pNR1 cells at CT16 (*p* = 0.0040, **Fig. 4B & D**), without influencing other time points. Following enucleation, both pERK (F[5], [29]  = 7.119, *p* = 0.0002) and pNR1 (F[5], [28]  = 4.84, *p* = 0.0026) remained rhythmic, and, in the absence of the CT16 peak, CT20 was now the time point with the highest number of core pERK (*p*  = 0.0015 vs. CT0, *p* = 0.0038 vs. CT4, *p* = 0.0006 vs. CT12 and *p* = 0.0006 vs. CT16) and pNR1 neurons (*p* = 0.0050 vs. CT0 and *p* = 0.0024 vs. CT4).

**Figure 5:**
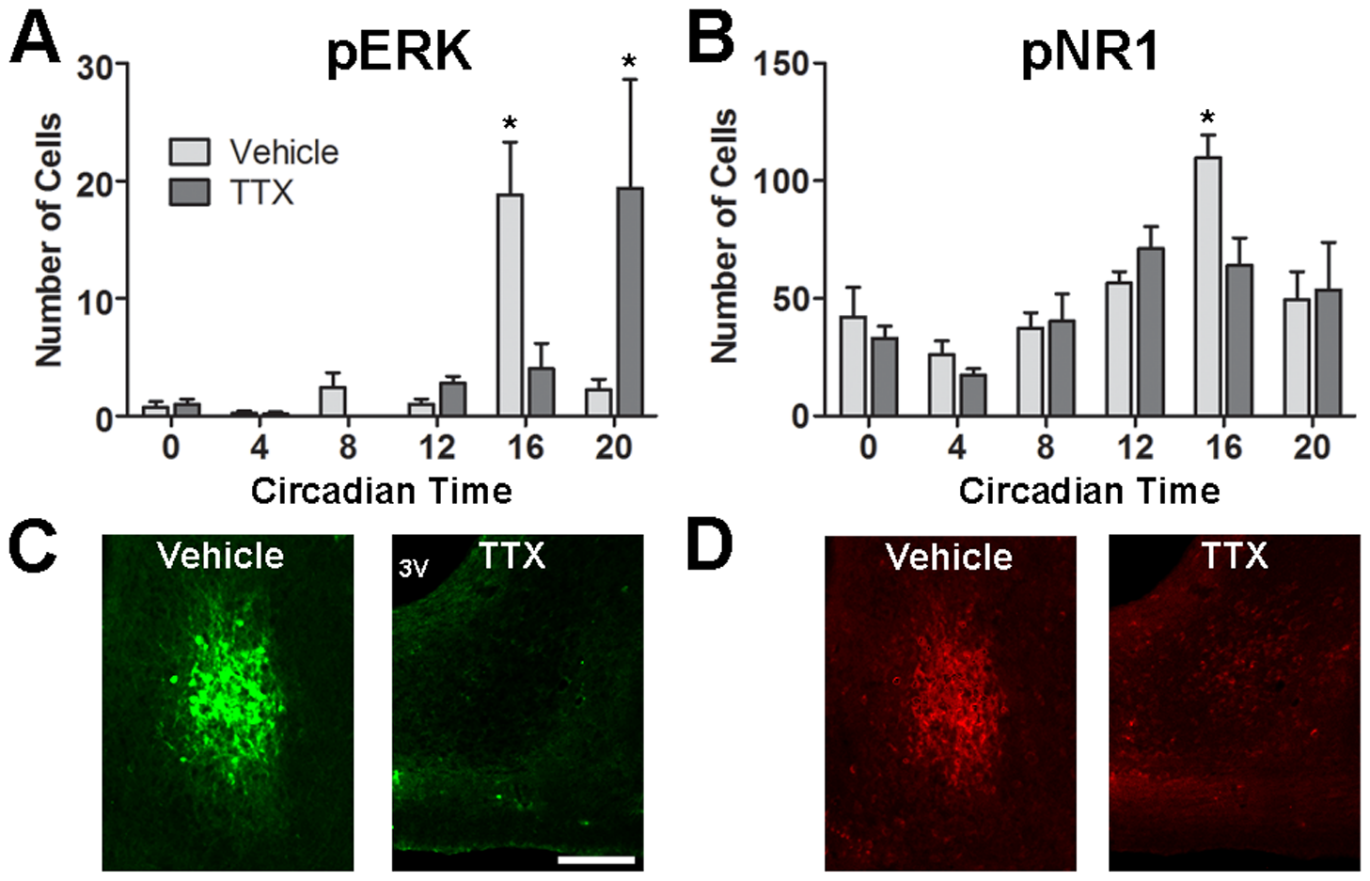
Effects of intraocular TTX administration on ERK and NR1 phosphorylation rhythms in the SCN core. Shown are the numbers of pERK (A) and pNR1 (B) cells in the SCN core across the circadian day in vehicle-treated and TTX-treated animals. Also shown are representative images of SCN core pERK (C) and pNR1 (D) immunostaining from vehicle-treated and TTX-treated animals at CT16. Scale bar  =  100 µm. * = p<0.05 vs. the experimental treatment at that time point.

To confirm that pERK and pNR1 rhythms in the SCN core are dependent on retinal neural output, hamsters were given intraocular injections of the voltage-gated sodium channel blocker TTX or vehicle. Analyses revealed an overall main effect of time (F[5], [49]  = 5.60, *p* = 0.0004) and a significant interaction (F[5], [49]  = 5.39, *p* = 0.0005) for the number of pERK cells, and a main effect of time (F[5], [50]  = 11.12, *p*<0.0001) and a marginally significant interaction (F[5], [50]  = 2.23, *p* = 0.0654) for pNR1. Similar to enucleation, intraocular TTX treatment significantly attenuated the peak in the number of pERK (*p* = 0.0073; **Fig. 5A & C**) and pNR1 cells (*p* = 0.0092; **Fig. 5B & D**) at CT16. In contrast to that observed for enucleation, TTX increased core pERK at CT20 (*p* = 0.0087) compared to vehicle.

To further establish that TTX transiently attenuated pERK and did not induce permanent ocular damage, an additional group of animals were injected with TTX and examined one day, two days, or one week later at CT16. Densitometry analysis revealed that pERK immunostaining was significantly decreased at days 1 (6.7 +/− 1.8% area above threshold; *p* = 0.0081) and 2 (13.9 +/− 7.9%; *p* = 0.0083) following TTX administration relative to vehicle-treated controls (58.2 +/− 7.2%), but returned to baseline levels by day 7 (76.2 +/− 17.0%). These observations, in addition to the pupillometry results (**Fig.1**), confirm that TTX transiently inhibited retinal output over the time course of our experiments.

### Enucleation and intraocular TTX administration do not influence SCN shell ERK and NR1 phosphorylation rhythms

**Figure 6:**
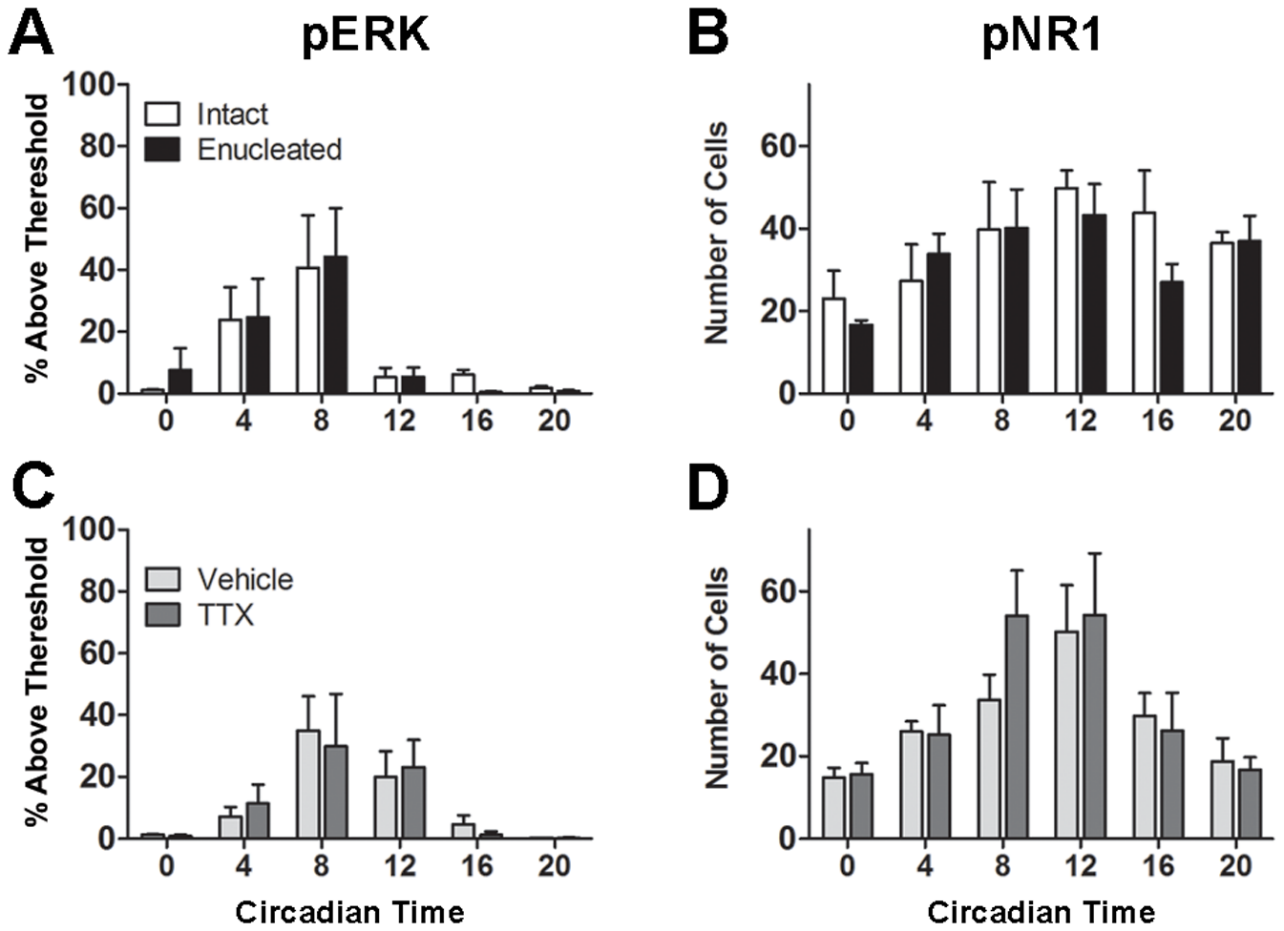
Effects of enucleation and intraocular TTX administration on pERK and pNR1 in the SCN shell . Shown are density (% area above threshold) of pERK (A & C) and the number of pNR1 cells (B & D) in the SCN shell across the circadian day in intact, enucleated, vehicle-treated, and TTX-treated animals.

As enucleation does not affect SCN shell pERK [9], the association between pERK and pNR1 rhythms following removal of the eye and TTX administration also was examined in this region. Overall analysis revealed a significant main effect of time on SCN shell pERK (intact and enucleated: F[5], [51]  = 7.96, *p*<0.0001; vehicle and TTX: F[5], [48]  = 6.09, *p* = 0.0002) and pNR1 (intact and enucleated: F[5], [59]  = 3.36, *p* = 0.0097; vehicle and TTX: F[5], [51]  = 6.52, *p*<0.0001). However, there was no effect of enucleation or drug treatment, nor were there significant interactions (**Fig. 6**). As observed previously [8], pERK immunostaining peaked at CT8 in intact animals (*p* = 0.0089 vs. CT20), and in the other treatment groups (enucleated: *p* = 0.0143 vs. CT0, *p* = 0.0073 vs. CT12, *p* = 0.0039 vs. CT16 and *p* = 0.0040 vs. CT20; vehicle-treated: *p* = 0.0084 vs. CT0, *p* = 0.0085 vs. CT16, and *p* = 0.0041 vs. CT20; TTX-treated: *p*<0.05 vs. CT20). The number of pNR1 cells showed marginal rhythmicity with significant peaks observed at CT12 for enucleated (vs. CT0, *p* <0.05) and vehicle treated-animals (*p* = 0.0094 vs. CT0, *p* = 0.0088 vs. CT20) but no significant peaks detected in intact or TTX-treated animals. The majority of shell pERK cells observed over all time points also colocalized with pNR1 (82% of 164 total pERK cells observed).

### MK-801 attenuates ERK and NR1 phosphorylation in the SCN core

**Figure 7:**
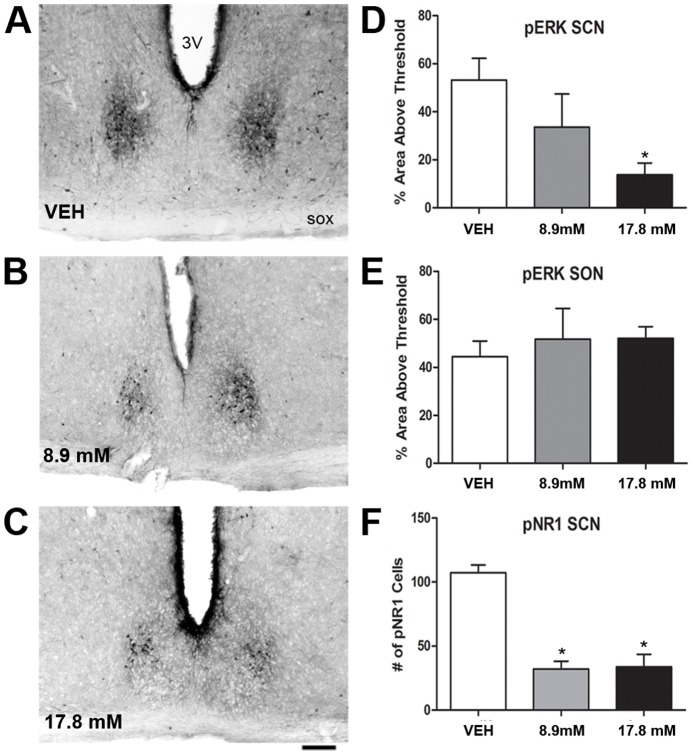
Effects of intracerebroventricular MK-801 administration on pERK and pNR1 in the SCN core and SON. A-C: Shown are representative images of pERK immunostaining in the SCN core from animals treated with vehicle (VEH), 8.9 mM MK-801, and 17.8 mM MK-801. Scale bar  =  100 µm. D-E: Densitometric quantification of the effects of MK-801 treatment on pERK immunostaining in the SCN and supraoptic nucleus (SON), respectively. F: Quantification of the effects of MK-801 treatment on the number of pNR1 cells in the SCN core. * = p<0.05 vs. VEH.

To determine if NMDA receptor activation is necessary for the peak in SCN core ERK phosphorylation, the NMDA receptor antagonist MK-801 was administered into the 3^rd^ ventricle at CT15. Analysis of pERK immunostaining at CT16 revealed a significant effect of treatment (F[2], [15]  = 4.19,, *p* = 0.0358) where the higher dose of MK-801 significantly decreased SCN core pERK compared to vehicle (*p* = 0.0151), and the low dose had an intermediate effect that failed to reach statistical significance (p = 0.1819, **Fig. 7A-D**). pERK immunostaining in the SON was unaffected by MK-801 administration (**Fig. 7E** **)**, suggesting that MK-801 acted locally in the SCN to exert its effects.

To confirm that MK-801 blocked NMDA receptor activation in the SCN core, the numbers of pNR1 cells were examined in MK-801 treated animals. Both doses of MK-801 significantly reduced NR1 phosphorylation as compared to vehicle treatment (F[2], [11]  = 27.82, *p*<0.0001; VEH vs. 8.9 mM: *p*<0.001; VEH vs. 17.8 mM: *p* = 0.0003; **Fig. 7F**).

### PACAP_6-38_ attenuates ERK and NR1 phosphorylation in the SCN core

**Figure 8:**
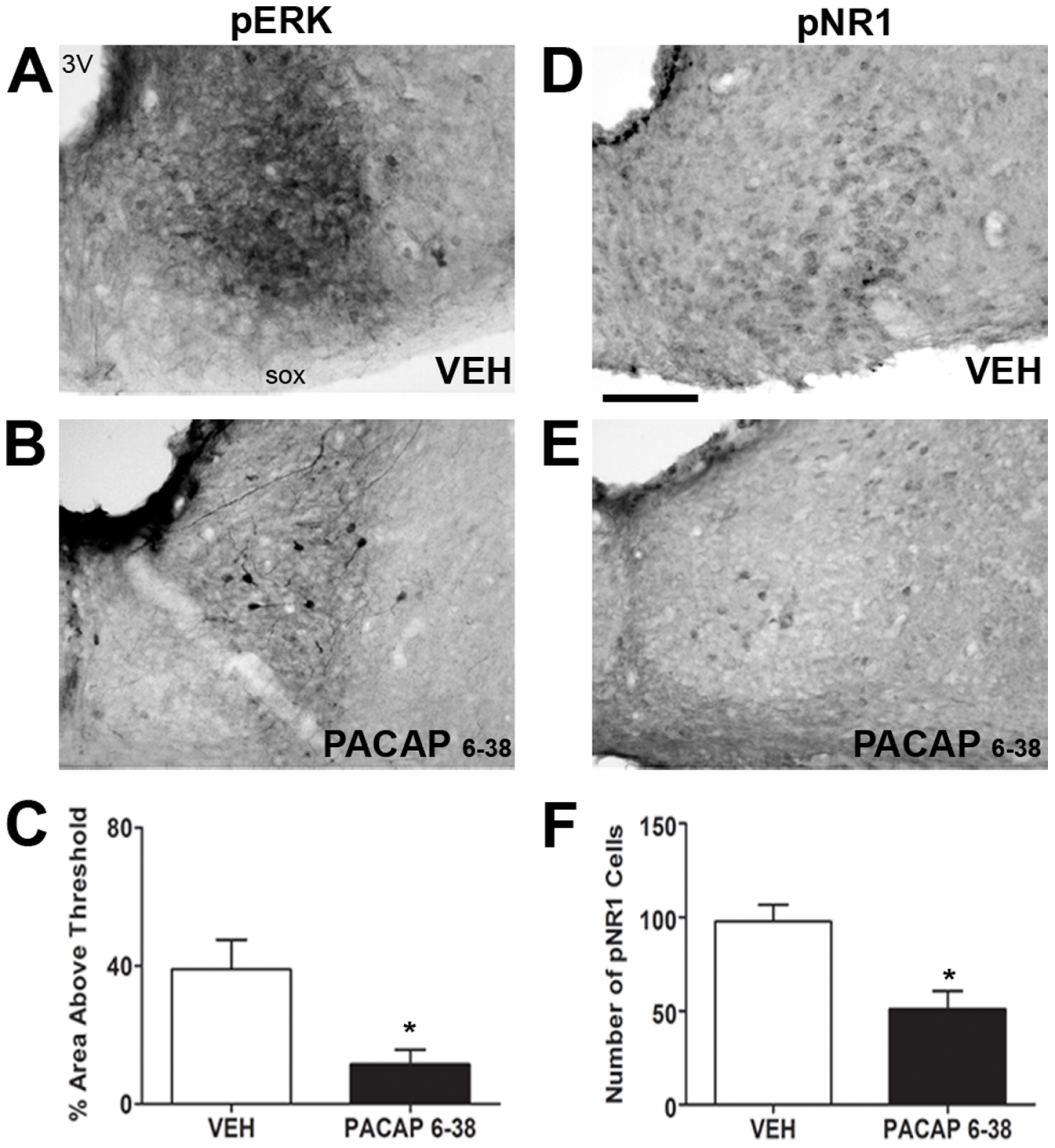
Effects of intracerebroventricular PACAP _6–38_ administration on pERK and pNR1 in the SCN core. Shown are representative images of pERK (A-B) and NR1 (D-E) immunostaining in the SCN core from animals treated with vehicle (VEH) or PACAP_6–38_. Also, shown is quantification of pERK (C) and pNR1 (F) immunostaining for each condition. * = p<0.05 vs. VEH. Scale bar  = 100 µm.

As PACAP is colocalized with glutamate in retinal terminals [21] and modulates NMDA receptor signaling [43], the role of PAC_1_ receptor activation in SCN core ERK phosphorylation was examined. pERK immunostaining in the SCN core was significantly attenuated by PACAP_6–38_ administration compared to vehicle (*p* = 0.0207, **Fig. 8A-C**). pERK immunostaining in the SON was unaffected by PACAP_6–38_ administration (41.7 +/– 4.0 vs 43.6 +/– 4.6).

To investigate whether PAC_1_ receptor-mediated signaling influenced NMDA receptor phosphorylation, the effect of PACAP_6–38_ administration on SCN core pNR1 immunostaining was examined. PACAP_6–38_ significantly attenuated the number of pNR1 cells in the SCN core compared to vehicle (p = 0.0105; **Fig. 8D-F**), indicating that PAC_1_ receptor activation contributes to NR1 phosphorylation.

## Discussion

**Figure 9:**
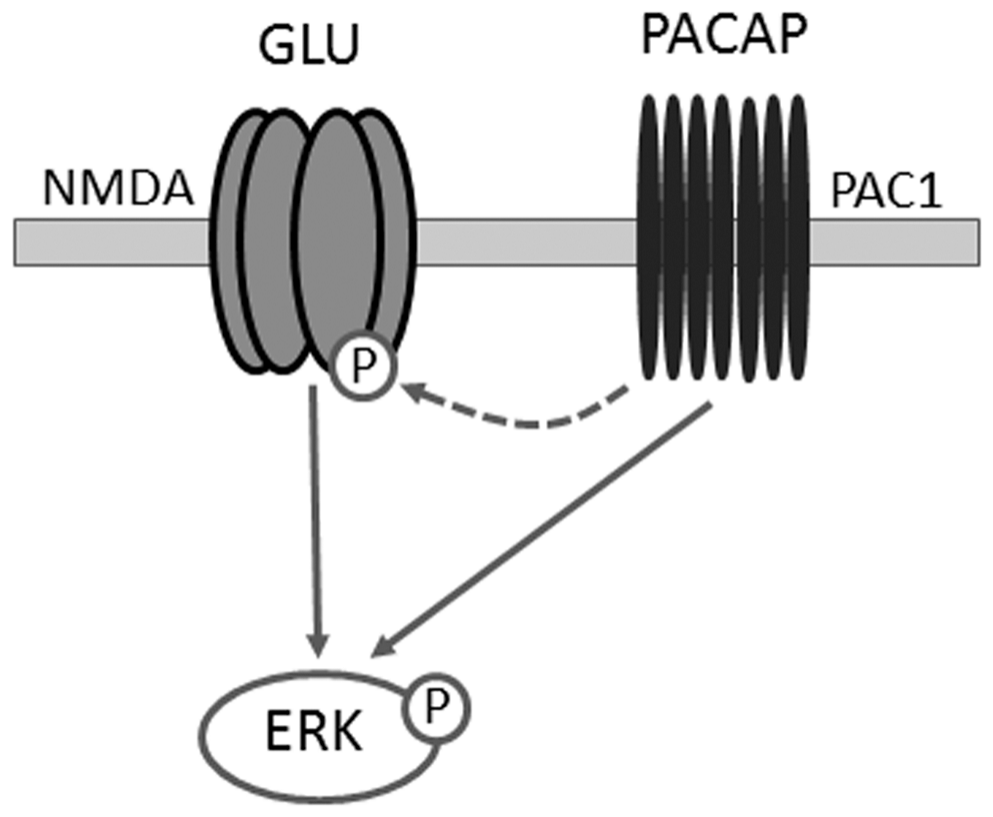
Induction of retinal-mediated SCN core ERK phosphorylation via NMDA and PAC_1_ receptor activation. Glutamate (GLU) and PACAP released from the retinohypothalamic tract act via NMDA and PAC_1_ receptors, respectively, to directly (solid lines) induce ERK phosphorylation (P). PACAP may also contribute to pERK indirectly (dashed line) via a potentiation of NMDA receptor signaling.

These results demonstrate that NMDA and PAC_1_ receptor signaling interact to induce retinal-mediated SCN core ERK phosphorylation that occurs in the absence of light. Rhythmic phosphorylation of the NR1 subunit of the NMDA receptor in the SCN core occurs in pERK-immunoreactive cells, shows a similar temporal profile to pERK, and, like pERK its rhythmic peak is dependent upon inputs from the eye. Moreover, administration of MK-801 dose-dependently attenuates the peak in the SCN core pERK rhythm. Thus, NMDA receptor activation contributes to retinal-mediated SCN core ERK phosphorylation. As PACAP is colocalized with glutamate in the RHT [21] and modulates NMDA receptor signaling [43], we hypothesised that this neuropeptide also contributes to retinal-induced pERK. Indeed, administration of a PAC_1_ receptor antagonist attenuated SCN core pERK and pNR1. Taken together, these results suggest that rhythmic ERK phosphorylation in the SCN core is induced by retinal-mediated NMDA and PAC_1_ receptor activation, with PAC_1_-mediated signaling cascades acting directly, and indirectly via a modulation of NMDA receptor signaling (**Fig. 9**). As the rhythm in SCN core pERK persists under constant darkness, these findings indicate that activation of NMDA and PAC_1_ receptors via retinal input during the night serves a function in the SCN distinct from their roles in photic clock resetting.

The current work also points to an endogenous rhythm in glutamate and PACAP release from retinal terminals that persists in the absence of light; a somewhat surprising conclusion given the extensive evidence implicating these neurotransmitters in photic entrainment [3], [24]. However, earlier microdialysis work in rodents has identified an endogenous rhythm in SCN glutamate levels, with a peak during the mid-dark period [44], [45]. The current data suggests that this peak in SCN glutamate may be driven, in part, via release from retinal terminals. What is perhaps more intriguing, is that nocturnal activation of NMDA and PAC_1_ receptors by retinal input does not result in circadian clock resetting. The reasons for this are unclear but may relate to differences in the magnitude of retinal input across these conditions or to a differential activation of SCN sub-regions. Photic stimulation results in relatively high levels of ERK phosphorylation over virtually the entire SCN [7], [8], [46], [47] whereas retinal-mediated pERK in the absence of light is confined to a relatively small portion of the dorsomedial caudal SCN. This spatial segregation occurs despite retinal innervation of all rostral-caudal levels of the rodent SCN [48], [49], [50], suggesting that a particular subset of the ipRGCs that comprise the RHT drives rhythmic ERK phosphorylation in the SCN core. Selective endogenous activation of these ipRGCs in the absence of light simply may not be sufficient to induce clock resetting.

The ocular-mediated rhythm in SCN core NMDA and PAC_1_ receptor activation implies the existence of a retinal circadian oscillator that regulates the output of ipRGCs that project to the caudal SCN core. Indeed, it is well-established that a self-sustaining circadian oscillator is located in the eye [51], [52], [53], [54], [55], and circadian clock gene expression has been observed in most retinal cell types, including ganglion cells [56], [57], [58]. However, it remains unclear if ipRGCs are intrinsically rhythmic or if rhythms in these cells are induced by oscillators located elsewhere in the retina. On one hand, pharmacologically isolated ipRGCs show a weak circadian rhythm in sensitivity to bright light *in vitro* [59], suggesting that these cells act as autonomous circadian clocks. However, degeneration of rods or cones, or of cells in the inner retinal layer, attenuates the circadian rhythm in melanopsin expression that has been observed *in vivo* [60], [61]. Hence, further work will be necessary to identify the locus of the retinal cellular oscillators necessary for the endogenous rhythm in input to the SCN.

In addition to decreasing the peak in SCN core pERK, administration of a PAC_1_ receptor antagonist decreased pNR1 in this region. Glutamate binding to the NMDA receptor can induce signaling cascades that activate PKA and PKC, resulting in phosphorylation of NR1 at Ser896 and Ser897 [62]. Thus, phosphorylation of the NR1 subunit may be used to index NMDA activation [36], [37], a conclusion supported further by our findings that SCN core pNR1 is attenuated by MK-801 administration. Taken together, these observations suggest that PAC_1_ mediated signaling cascades act directly, and indirectly via a potentiation of NMDA receptor signalling, to induce SCN core pERK. Indeed, it is well-established that PACAP can modulate SCN NMDA-mediated responses via both pre- and post-synaptic mechanisms [28], [32], [63], [64], [65]. However, as PAC_1_ receptor activation also can stimulate PKA and PKC [66], [67], and thus presumably directly phosphorylate the NR1 subunit, further work will be necessary to confirm a modulatory influence in this context, and to characterize the mechanisms of interaction.

We have previously reported that enucleation attenuates SCN core ERK phosphorylation in the rodent SCN [9] and confirm the results here. Consistent with the disruption of pERK and pNR1 rhythms by enucleation, intraocular TTX administration decreased peak ERK and NR1 phosphorylation in the SCN core. Thus, retinal neural signaling is necessary for the peak in pERK and pNR1 in the core region. The ability of TTX to block the nighttime peak of pERK and pNR1 also demonstrates that the enucleation effect is not due to secondary changes in the SCN following the loss of retinal afferents, nor is it via disruption of a humoral signal from the eye. However, unlike the effect of enucleation, intraocular TTX administration significantly increased pERK in the SCN core during the late subjective night. While the reasons for this discrepancy are unclear, there are several potential explanations. First, TTX may induce a phase delay of the retinal oscillator, thus shifting the peak to a later time. This seems unlikely as TTX administration does not induce a coincident increase in pNR1 at CT20. It is also possible that this discrepancy is due to a difference in the time course of ocular inhibition. That is, the TTX-induced increase in SCN core pERK at CT20 may be a transient compensatory response to the loss of retinal input that is absent in enucleated animals over the long term. Examining the short-term effects of enucleation may help to address this possibility. Finally, it may be argued that ERK phosphorylation during the late night is induced via extra-retinal inputs and that TTX somehow increases this activity. This is supported, in part, by the observation that a small number of core pERK cells remain at CT20 following enucleation.

Consistent with our previous findings [9], pERK in the SCN shell was unaffected by enucleation. The current results extend these findings to intraocular TTX injections and also show that NMDA receptor activation in this region is largely unaffected by removal of the eye or TTX. Although only marginal rhythmicity in pNR1 was seen in the SCN shell, pERK and pNR1 are extensively colocalized in this area. Thus, it appears that glutamate release from extra-retinal sources contributes to rhythmic ERK phosphorylation in the shell region. As circadian rhythms in SCN glutamate release have been observed *in vitro* [68], it is possible that the rhythmicity in shell ERK and NR1 phosphorylation is mediated by glutamate released from within the nucleus. Activation of ERK in the shell may represent a synchronizing signal among oscillator neurons as inhibition of pERK *in vitro* attenuates rhythms in SCN neuronal firing, and clock gene and vasopressin expression [69], [70].

The functional role of retinal input to the SCN in the absence of light remains unclear. Certainly, it is not involved in conveying environmental timing information to the nucleus. However, it is possible that this input modulates the process of photic entrainment. Indeed, retinal-mediated pERK cells are extensively colocalized with GRP (in hamsters: Webb and Lehman, unpublished observations; in rats:[71]), a neuropeptide widely implicated in the processing of photic information [72], [73], [74], [75], [76], [77], [78], [79]. As light exposure has been reported to have enduring effects on the membrane properties of GRP neurons [80], it is conceivable that retinal input in the absence of light may have a similar long-term modulatory influence on this cell population and thus alter any subsequent photic response. Given the timing of the peak in core pERK and pNR1 relative to the photic phase response curve [81], it is tempting to postulate a link between the activation of GRP or other cell populations by the eye and the transition at CT16 from photic phase delays to advances. Further work will be necessary to examine these possibilities.

Enucleation has also been reported to result in a broader range of free-running periods [82], suggesting that precise circadian period determination may be a function of coupling between the retinal and SCN oscillators. Consistent with this notion, photoreceptor ablation or degeneration [83], [84] and disruption of PACAP signaling [85] are associated with an altered free-running period. These findings point to an influence of retinal input on circadian period and the current results suggest that glutamate and PACAP acting upon GRP cells may be part of this mechanism. Indeed, GRP has recently been reported to promote SCN cellular coupling *in vitro* [86]. However, any effects of retinal input on SCN period are likely minimal, as the period of *period1* expression in cultured SCN explants, while slightly more variable, is essentially equal to the locomotor activity rhythm in the intact animal [87]. Hence, the observed effects of retinal manipulations on circadian period are likely mediated by extra-SCN sites involved in the expression of behavioral rhythms.

In sum, the current work suggests that rhythmic phosphorylation of ERK in the SCN core, as seen in constant darkness, is due to retinal-mediated activation of NMDA and PAC_1_ receptors. These data point to a previously unsuspected role for the eye in modulating SCN function, and a role for NMDA and PAC_1_ receptor activation in the SCN apart from the induction of photic clock resetting. Although further work is needed to delineate the precise role of this rhythmic retinal input, potential functions include regulating the phase responsiveness of the SCN to light at different times during subjective night, or synchronization among SCN neurons resulting in a more precise clock period. The current results also reinforce the view of the SCN as a heterogeneous structure comprised of multiple tissue-level oscillators [5] and suggest that its interactions with the eye, even in the absence of light, may be of key importance in determining its properties.
